# Great tits feed their nestlings with more but smaller prey items and fewer caterpillars in cities than in forests

**DOI:** 10.1038/s41598-021-03504-4

**Published:** 2021-12-17

**Authors:** Csenge Sinkovics, Gábor Seress, Ivett Pipoly, Ernő Vincze, András Liker

**Affiliations:** 1 Behavioural Ecology Research Group, Center for Natural Sciences, University of Pannonia, Veszprém, PO Box 1158, 8210 Hungary; 2 MTA-PE Evolutionary Ecology Research Group, University of Pannonia, Veszprém, PO Box 1158, 8210 Hungary; 3 Theoretical Population Ecology and Evolution Group, Department of Biology, Lund University, Lund, Sweden

**Keywords:** Zoology, Ecology, Urban ecology

## Abstract

Rapidly increasing urbanisation is one of the most significant anthropogenic environmental changes which can affect demographic traits of animal populations, for example resulting in reduced reproductive success. The food limitation hypothesis suggests that the shortage of high-quality nestling food in cities is a major factor responsible for the reduced reproductive performance in insectivorous birds. To study this explanation, we collected data on the parental provisioning behaviour of urban and forest great tits (*Parus major*) in three years that varied both in caterpillar availability (the main food of great tit nestlings) and in reproductive success of the birds. In all years, urban parents provisioned caterpillars in a smaller proportion to their nestlings, but the total amount of food per nestling (estimated by the volumes of all prey items) did not differ between habitats. In the two years with much lower reproductive success in urban than forest habitats, urban parents had higher provisioning rates, but provided more non-arthropod food and brought smaller prey items than forest parents. In the year with reduced habitat difference in reproductive success, urban parents were able to compensate for the scarcity of caterpillars by provisioning other arthropods rather than non-arthropod food, and by delivering larger preys than in the other years. Specifically, in this latter year, caterpillars provisioned by urban pairs were cc. twice as large as in the other two years, and were similar in size to caterpillars provisioned in the forest broods. These results show that although urban great tit parents can provide the same quantity of food per nestling as forest parents by reducing their brood size and increasing the per capita feeding rates for nestlings, they cannot compensate fully for the scarcity of high-quality preys (caterpillars) in poor years. In some years, however, favourable conditions for urban caterpillar development can greatly reduce food limitation in cities, allowing urban birds to achieve higher reproductive success. We suggest that urban green areas designed and managed in a way to facilitate conditions for phytophagous arthropods could improve habitat quality for urban birds.

## Introduction

As the human population is growing, habitat urbanisation has become one of the most important factors posing threats to natural habitats, biodiversity and wildlife populations^1,2^. Insectivorous birds provide a particularly well-documented example, because several studies showed that their populations often have inferior reproductive success in urban compared to more natural habitats^3–12^. These detrimental effects of urbanisation are often explained by the food limitation hypothesis, which proposes that the low availability of high-quality food for offspring is a major driver of the reduced reproductive success in urban areas^13–16^.

Lepidopteran larvae (caterpillars) are the primary nestling food for many insectivorous bird species in temperate deciduous forests^17,18^. Caterpillars provide a high-quality food source because they are rich in proteins^19^ as well as carotenoids which have essential functions as antioxidants, immunostimulants, and pro-vitamins in birds, and also influence plumage colour signalling^20–23^. The abundance of caterpillars—similarly to many other insects—has been dramatically declining in urban areas compared to more natural habitats, which may be a major contributor to the food limitation of insectivorous animals in cities and towns^15,24–26^.

Urban breeding birds can adapt to this reduced natural food supply in at least three mutually non-exclusive ways. First, the negative effects of food limitation on nestling development and survival can be mitigated by laying smaller clutches. This strategy can reduce the detrimental effects of food limitation as fewer nestlings require less amount of food. According to this expectation, studies commonly find smaller clutches in urban than in non-urban populations^8,27^.

Second, urban parents may increase their chick-feeding activity to provide a sufficient amount of natural food for their nestlings if large prey items are scarce, for example, due to the smaller size of urban arthropods^28–30^. Supporting this scenario, smaller food items were reported in urban than non-urban environments in the nestling diet of blue tits^31,32^ (*Cyanistes caeruleus*) and house sparrows^5^ *(Passer domesticus),* and an increased chick-feeding frequency was measured in urban relative to more natural habitats, for example, in great tits^33,34^ and blue tits^31^. Furthermore, to keep up the rate of nestling food deliveries urban parents may also have to fly further from their nest ^32^, similarly to what was found in blue tits breeding in good versus poor habitats in more natural environments^35^. Note, however, that a higher provisioning rate of small preys may not necessarily compensate for the reduced amount of large arthropod prey items in nestlings’ diet. A study in urban house sparrows showed that the frequency of large arthropod prey items in the diet, and not parents’ feeding rates per se, predicted brood size and nestling body mass^36^, which can be related to the size-dependent variation in arthropods' calorific or nutritional content. For example, Lease and Wolf^37^ found that the lipid content of arthropods shows an isometric scaling relationship with respect to their body mass, resulting in a proportionately larger quantity of nutrients (e.g. lipids) in large compared to smaller arthropods.

Third, urban parents might try to compensate for the relative scarcity of high-quality diet components, like caterpillars and other arthropods, by provisioning anthropogenic food items (e.g., garbage, bird food) to their offspring. Such altered nestling diet has been reported in several urbanized bird species, including blue tits^31,32^, great tits^38^, common starlings^39^ *(Sturnus vulgaris)* and Florida scrub-jays^40^ *(Aphelocoma coerulescens)*. Although anthropogenic food sources are typically abundant and easily accessible in urban environments^41^, their nutritional composition is likely to be inadequate to support optimal nestling development^39,42^. Indeed, the most common food items at bird tables (e.g. birdseed, tallow, peanut cakes, or bread crumbs) are rich in fat and carbohydrates, making them a good source of energy for adults in winter, but typically contain low amounts of nutrients like protein or calcium^43,44^ which are fundamental growth-limiting factors for nestlings in spring^45^. Thus, the altered palette of nestling food may result in nutrient deficiency rather than caloric restriction for urban nestlings. In line with this assumption, a number of studies reported links between reduced breeding success and increased amount of plant material^46^, fat^47^, or human refuse^39^ in nestlings’ diet in birds that normally rely on arthropod-based food.

Based on the above findings, the availability of different, natural food types is a key factor for insectivorous birds that determines the success of nestling rearing. However, some long-term studies highlighted that both the food availability during breeding season and the breeding success of birds may vary significantly not only between habitats but also between years^15,26,48^. For example, in an earlier study we analysed caterpillar biomass and breeding success of great tits in urban and forest habitats in 4 years (2013–2016)^15^ and in line with other studies^26,31^, we found strongly decreased caterpillar biomass in the canopies of urban relative to forest trees (the yearly mean ± SE of hourly caterpillar biomass (mg/h) during the nestling rearing period was ranging from 1.67 ± 0.1 to 5.86 ± 0.54 in the urban sites, and from 50.09 ± 6.25 to 122.96 ± 13.93 in the forest sites, see Fig. 3. in the cited publication^15^). In the same study, great tits laid smaller clutches and produced fewer and smaller fledglings (as measured at the age of ringing, 15 ± 1 days after hatching) in urban study sites, although the extent of habitat difference varied between years. Remarkably, in that specific year (2014) when we detected the smallest magnitude of habitat differences in caterpillar biomass we also found the smallest habitat differences in breeding success^15^. These results along with a recent experimental study^16^ strongly suggest that in our study system the most important driver of the low reproductive success of urban great tits is food limitation during brood rearing. However, we have not explored yet how low caterpillar availability in the environment affects nestling diet and parental provisioning in these populations, which ultimately determines the influence of food limitation on the development and survival of nestlings in urban broods.

The aim of the present study was to compare the composition and the amount of nestling food between urban and forest habitats. To do this, we analysed video recordings of the parents’ feeding behaviour in two urban and two forest great tit populations and determined the prey type and volume of the food items brought to the nestlings. To make our results generalizable, we involved a large number of broods (153 broods and more than 3000 feeding events) and conducted the study over three breeding seasons (2014–2016) that showed marked between-year differences in both the biomass of canopy-dwelling caterpillars and the great tits’ reproductive success^15^ (see above). In urban compared to forest great tit broods, we predicted (i) a decreased proportion of caterpillars and increased proportions of other arthropods and/or non-arthropod (e.g. anthropogenic) food items in the nestling diet, (ii) smaller size of prey items provisioned (including both caterpillars and other food items), and (iii) a lower amount of food per nestling. Furthermore, we also predicted to find (iv) the smallest habitat difference in the composition and/or amount of nestling diet in the year (2014) when we detected the smallest habitat differences in both the caterpillar biomass (in tree canopies) and the birds’ reproductive success^15^.

## Methods

### Ethical statement

All procedures were in accordance with Hungarian laws, and adhered to the ASAB/ABS guidelines for the use of animals in behavioural research and teaching. The use of animals in our studies were approved by the National Scientific Ethical Committee on Animal Experimentation (permit numbers: PE-06/KTF/997–8/2018, FPH061/1329–5/2018). Permits to study protected species and access to protected areas were provided by the Middle Transdanubian Inspectorate for Environmental Protection, Natural Protection and Water Management (permit numbers: 31559/2011 and 24,861/2014).

### Study sites and data collection

We studied great tits at two urban and two forest sites as part of a long-term study conducted in Hungary (Supplementary Material, Fig. S1). The urban study sites are located in Veszprém (47°05′17″N, 17°54′29″E) and Balatonfüred (46°57′30″N, 17°53′34″E), where the artificial nest boxes are placed in public parks, a cemetery, and university campuses where vegetation consists of both native and exotic species. One of the forest study sites is located at Vilma-puszta (c. 3 km from the edge of Veszprém; 47°05′06.7″N, 17°51′51.4″E) dominated by downy oak (*Quercus pubescens*) and South European flowering ash (*Fraxinus ornus*). The other forest study site (47°06′39.75″N, 17°41′17.94″E) is located 3 km away from the nearest human habitation, Szentgál, and c. 20 km away from Veszprém. This study site is dominated by beech (*Fagus sylvatica*) and hornbeam (*Carpinus betulus*). The nest boxes were checked at least twice a week throughout each breeding season (March-July) to determine laying dates, clutch sizes, hatching dates, and brood sizes of breeding great tits.

In three years (2014, 2015, and 2016) we video-recorded the nestling feeding behaviour of parent birds at the nest to collect data on the composition and amount of nestling diet. We collected one 60-min long video sample per brood, because this sampling length was suggested sufficient to characterize variation in parental provisioning in great tits^49^. Video-recordings started between 8:00 and 18:15 (mean ± SE, forest: 11:57 ± 00:14, urban: 12:28 ± 00:18) when nestlings were about 10 days old (range: 8–12 days, mean ± SE forest: 9.82 ± 0.11, urban: 10.02 ± 0.1; day of hatching = day 1) because nestlings’ food demand is the highest at this age^50,51^. The camera (GoPro HD HERO 2 or HERO 3) was placed into a black plastic box outside the nest box (about 15 cm from the entrance hole) to minimize any possible disturbance due to its installation (Fig. S2). The camera box is a constant part of our nest boxes, so breeding birds are familiar with them and the presence of cameras in the boxes does not influence the parents’ behaviour^52^. The nest boxes also had a small wooden shelf right below the entrance hole where parent birds could land and often pause for a few seconds before entering the nest with the delivered food item (Fig. S2). Before placing the camera into the plastic box for the recording, we counted the number of nestlings in the nest. We never captured or ringed parent birds or measured and ringed nestlings before the video-recordings because these processes can affect parental provisioning behaviour^52^. During the three study years, we collected video-recordings for 153 broods (2014: 30 and 20; 2015: 20 and 27; 2016: 27 and 29 forest and urban broods, respectively). Finally, we also collected hourly data of the ambient air temperature for each study site using our own on-site weather stations.

### Variables extracted from the video recordings

We included videos in our analyses only from the annually first broods where both parents took part in nestling provisioning (i.e. both parents appeared on the video or were observed at or captured on the nest later during the same breeding event). We regarded a brood as the first breeding attempt of a pair if it was initiated before the laying date of the earliest known second brood at that study site and year by an individually identifiable female that successfully raised her first brood (i.e. fledged at least one young). We excluded 12 (7.3%) video-recorded nests from the analyses where one of the parents was never observed. We collected detailed data from the video-recordings using VLC Media player^53^ (v. 2.2.0.) which allows slow-motion and frame-by-frame playback. We calculated six variables to describe the composition and amount of nestling food separately for each brood (Table 1).

**Table 1:** Variables used in the study to characterise nestling diet and food provisioning by parent great tits.

Variable	Categories / Calculation	Sample size, forest versus urban
(1) Prey type*	caterpillar, other arthropod, non-arthropod	1198 versus 1053 prey items
(2) Number of feeding visits	number of feeding visits during the 60-min recording	77 versus 76 broods
(3) Feeding rate	$\frac{\text{number of feeding visits}}{\text{number of nestlings}}$	77 versus 75 broods
(4) Average prey volume**	$\frac{\text{sum of the volume of measured prey items}}{\text{number of measured prey items}}$	49 versus 43 broods
(5) Hourly prey volume	$\frac{\text{average prey volume}*\text{number of feeding visits}}{\text{number of nestlings}}$	49 versus 42 broods
(6) Caterpillar volume	${\mathrm{\pi l}(0.5\mathrm{w})}^{2};$ l: caterpillar length, w: caterpillar width	855 versus 568 caterpillars

Data to calculate these variables were collected from 60-min video recordings conducted at each nest. Sample sizes differ between variables due to lack of information on the type of data necessary for the calculations or because the calculation was restricted to a subset of the broods (see Methods for explanations).

*Feeding events with unidentified prey items (n = 955, forest: 374, urban: 581 prey items) were excluded from the analyses.

**Volume was calculated for all prey items using the same formula given for caterpillar volume.

For each parental visit, we determined the (1) prey type, categorized as ‘caterpillar’, ‘other arthropod’ (e.g. spiders, mosquitos), ‘non-arthropod’ food item (mainly seeds). We categorized a prey item as ‘unidentified’ when it was not properly visible from the video recordings hence cannot be allocated to one of the above described three food type categories (either because the parent bird entered the nestbox too fast or the prey item was ‘smashed’). For each brood, we determined the (2) number of feeding visits (both parents combined) during the 60-min observation and calculated (3) feeding rate, as the number of feeding visits divided by the number of living nestlings (counted before the recording) for each brood. To quantify the amount of nestling food we estimated prey volume for each prey item following the method of Sinkovics et al.^54^ We measured the length and width of each prey item and calculated the volume assuming they had the shape of a cylinder. We used the entrance hole as a size reference (see the Supplementary Methods for a detailed description). We were able to estimate the volume for 63.8% of the delivered prey items (62.5% of prey items in urban and 65.3% in forest broods). From these prey volume measurements, we calculated the (4) average prey volume for each brood which refers to the volume of food delivered to the nest per feeding event. We also calculated the (5) hourly prey volume per nestling for each brood, by multiplying the average prey volume with the number of feeding visits and divided by the number of nestlings, which estimates the total amount of food that one nestling received during the 60-min observation. Finally, (6) individual caterpillar volume was also a separate variable, which we determined for each caterpillar specimen we were able to estimate from the videos (Table 1). We detected a total of 35 visits (1.2% of the total number of confirmed feeding visits) when parents entered the nestbox without prey (called ‘prey-free’ visits) and excluded these from all calculations. Besides, there were 259 visits, when a parent bird moved so fast across the screen that we did not see whether it had any prey in its beak (‘uncertain events’). These uncertain events were counted as feeding events with ‘unidentified’ prey items because ‘prey-free’ visits were very rare.

### Statistical analyses: the general scheme of modelling approach

**Figure 1:**
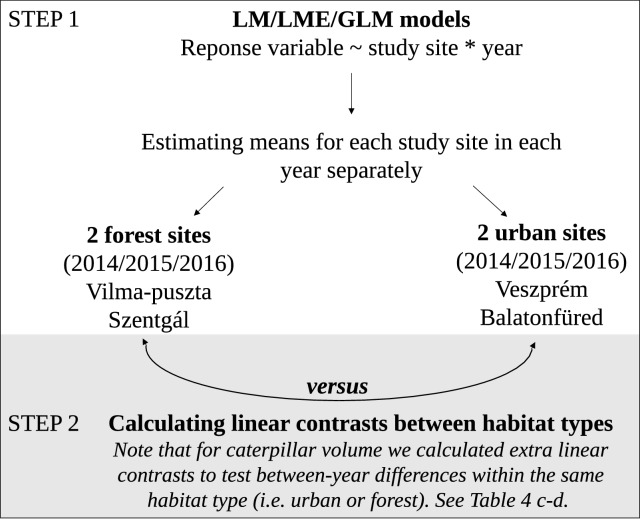
Schematic illustration of the statistical analysis process. In Step 1, we built a separate statistical model for each dependent variable. In Step 2, we tested habitat (forest vs. urban) difference in the dependent variable using linear contrasts from the model built in Step 1.

All statistical analyses were conducted using R statistical software^55^ (version 3.4.3.) using the following packages: “nlme”^56^, “emmeans”^57^, “MASS”^58^, “car”^59^, and “multcomp”^60^. To test our specific predictions for the effects of habitat and year on the composition and amount of nestling diet (see Introduction) we conducted pre-planned pairwise comparisons as suggested by Ruxton & Beauchamp^61^. In general, our data analysis consisted of two main steps (see a graphical illustration of the process in Fig. 1).

First, we constructed separate statistical models for each response variable. These models always included the study site (4 sites), year (3 years), and the study site × year interaction as predictor variables. We also tested the effects of the following potential confounding variables: ambient air temperature at the start of the observation, date, and time of the day at the start of the recording. Time of day was categorized as a three-level factor: between 8–11 h (n = 60 broods: 33 forest and 27 urban broods), 11–14 h (n = 59 broods: 31 forest and 28 urban broods), 14–18:15 h (n = 33 broods: 13 forest and 20 urban broods). Note that, because the onset of breeding season varied between years and sites, we mean-centered the date separately for each site and year combination, to express the relative dates of video recordings for each population within each year. This transformation reduced the multicollinearity of the date variable with year and site in the models and also controlled for possible seasonal effects in ‘early’ and ‘late’ first broods more precisely than the calendar date. Because none of these potential confounding variables had a significant effect in the models (see Tables S1, S2, S3 for the results of the extended models), in the second step of the analyses (pre-planned pairwise comparisons, see below) we used the results of models including only site, year, and site × year interaction.

Because for 12 pairs we had more than one video-recordings (from two of the three years), for each response variable we also built linear-mixed-effect models using pair ID as a random factor (the models were otherwise identical to that described above). We then compared each model pair (i.e. with and without the random factor) using likelihood ratio tests to see which model has a better fit. We found no statistically detectable differences between the model pairs (ΔAIC ≤ 2 and p > 0.779) indicating that inclusion of pair ID as random effect did not improve model fit, so we ran our models without pair ID. The models we built for each specific response variable were as follows.

### Analyses of the composition of nestling diet

To analyse the composition of the nestling diet we built generalized linear mixed models (GLMM) with binomial error distribution and “logit” link formula. We used brood ID as random factor to control for repeated feeding events by the same pair during 60-min. We built two models: the first model investigated the proportion of caterpillars in the nestling diet, thus we used the proportion of caterpillars as the response variable (coded as: caterpillars = 1, all other identified food items = 0). In the second model, we investigated the composition of the non-caterpillar part of the nestling food, and the response variable was the proportion of other arthropods (spiders, etc.; coded as: other arthropods = 1, non-arthropod prey items = 0). Note that in these two analyses we only included the identified prey items, i.e. we analysed only the identified fraction of the nestling diet (categorised as ‘caterpillar’, ‘other arthropod’, ‘non-arthropod; n = 2251; forest: 1198, urban: 1053 prey items), whereas the unidentified prey items were excluded from the models (n = 955, forest: 374 , urban: 581 prey items).

### Analyses of the amount of nestling food

To investigate differences in the amount of nestling food, we built separate linear models for the following four response variables: number of feeding visits, feeding rate, average prey volume, and hourly prey volume. In the models of the number of feeding visits, we could include all observations (n = 153 broods: 77 forest and 76 urban broods), while we had to exclude one urban brood from the feeding rate model due to missing information on brood size (Table 1). For average prey volume and hourly prey volume, we used only a subset of the observed broods (for average prey volume: n = 92 broods: 49 forest and 43 urban and broods, for hourly prey volume: n = 91 broods: 49 forest and 42 urban broods; Table 1.) which fulfilled the following two conditions: (1) we were able to estimate the volume of at least four prey items during the 60-min recording, and (2) at least 60% of the prey items had volume estimations. We used these two criteria because for some nests we were able to determine prey volume only for a small fraction of prey items, so the estimation of the average and hourly prey volumes would have been highly uncertain and/or biased in such cases. We tested several other thresholds for these inclusion criteria (minimum 4 prey volume estimation and at least 50%, 60%, 70%, 80% of prey items had volume estimation, or minimum 6 prey volume estimation and at least 50%, 60%, 70%, 80% of prey items had volume estimation) but the direction and magnitude of the site/habitat/year differences were not influenced by applying stricter conditions (which, however, resulted in much more reduced sample sizes). Thus we chose the above thresholds because those provided a balance between reducing uncertainty and still allowing the use of appropriate sample sizes for statistical analyses.

To investigate differences in caterpillar volume between study sites and years, we built a linear mixed-effect model (LME) where the response variable was caterpillar volume (i.e. estimated volume of individual caterpillars, n = 1423, Table 1). We included the brood ID as a random factor because several caterpillars from the same 60-min-observations were included. We applied the cube root transformation to caterpillar volume to meet the assumptions of the models.

### Comparisons between urban and forest habitats and among years

In the second step of the analyses (Fig. 1.), we conducted pre-planned pairwise comparisons to test our specific predictions for the effects of habitat and year on the composition and amount of nestling diet. To do this, we calculated marginal means from the models described above for each of our study sites in each year using “emmeans” package^57^. Then, we compared the two habitat types by calculating and testing the difference of these marginal means between the two forest sites versus the two urban sites for each year (Fig. 1). All these differences were derived from the parameters and associated errors estimated by each model as linear contrasts of least‐squares means^57^. For the LME model of caterpillar volume, we calculated two additional sets of linear contrasts as follows: (1) between-year comparisons within the forest habitat and (2) between-year comparisons within the urban habitat (i.e. 2014 vs. 2015, 2014 vs. 2016 and 2015 vs, 2016 for both habitats). Please note that a significant site effect (or site × year interaction) detected in the linear models does not necessarily result in a significant habitat effect (or different habitat effects in different years) in the contrast analyses because these effects may be inconsistent between the two sites in the same habitat. Conversely, weak and non-significant site effects (or its yearly variation) can produce a detectable difference in the habitat comparisons.

We used this linear contrast approach rather than including habitat type as a fixed effect and site as a random effect in the models because variance estimations of random effects with few levels (only four in our case) are unreliable^62,63^. We applied the false discovery rate (FDR) method for correcting *P*-values for multiple comparisons. We checked the statistical assumptions for each model by visually examining their residual plots^64^. We define the statistical significance level at 0.05 and refer to results where 0.05 < P < 0.1 as marginally non-significant.

## Results

### Composition of nestling diet

**Figure 2:**
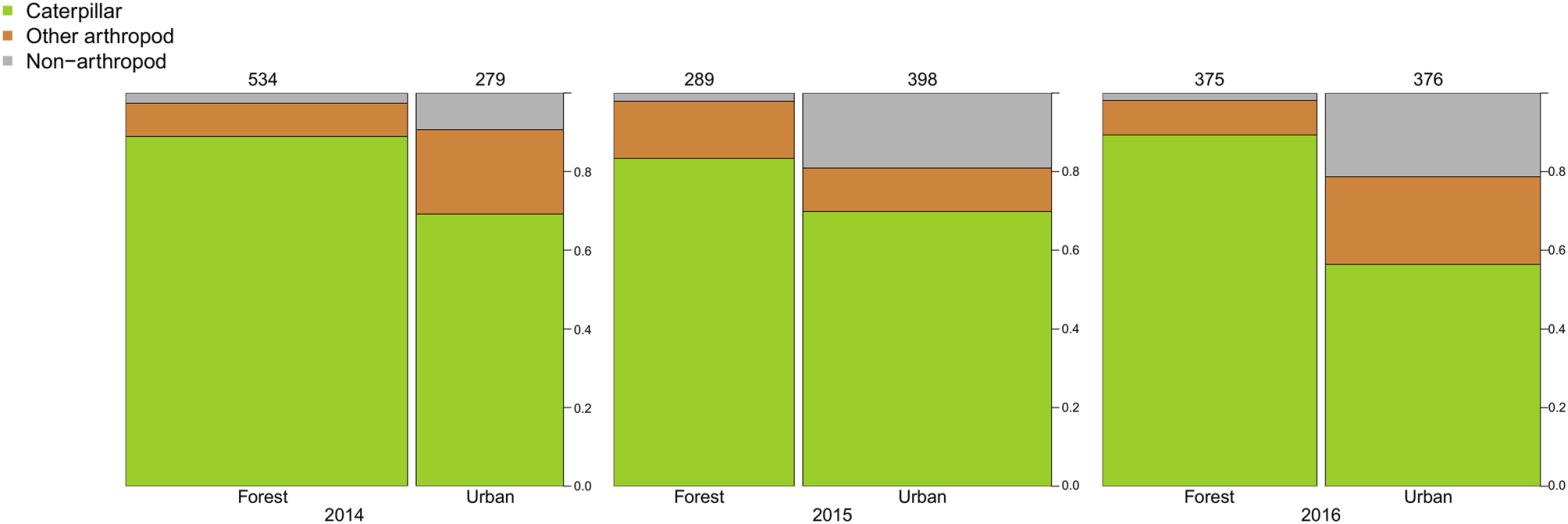
Proportions of different prey types (caterpillar, other arthropod, and non-arthropod) provisioned to great tit nestlings for each year and habitat type. The numbers of identified prey items are indicated above each column and are proportional to the width of the columns.

In total, we recorded 3206 feeding events and were able to assign 2251 prey items into the three analysed prey type categories (caterpillar, other arthropod, non-arthropod). The proportion of caterpillars in the nestling diet showed significant differences between study sites and we also found a significant site × year interaction (Table 2a). The linear contrasts indicated that, in each year, forest great tit nestlings received a higher proportion of caterpillars than urban ones, although in 2015 the habitat difference was marginally non-significant (forest vs. urban: 2014: 89% vs. 69.2%; 2015: 83.4% vs. 69.8%; 2016: 89.3% vs. 56.4%; Fig. 2, Table 2b). In the case of the non-caterpillar fraction of the nestling food, we found that the proportions of the two food categories (i.e. other arthropod prey vs. non-arthropod prey) differed both between sites and years, although the site × year interaction was not significant (Fig. 2, Table 2a). The linear contrasts indicated that in 2015 and 2016 urban nestlings received lower proportions of non-caterpillar arthropods compared to forest nestlings, but we found no such habitat difference in 2014 (Fig. 2, Table 2b).

**Table 2:** Comparisons of the composition of nestling diet between sites, habitats, and years.

(a) GLM models				(b) Linear contrasts between urban and forest habitats in each year		
Predictors	DF	χ2	*p* value	year	contrast ± SE	adjusted* p* value
**Caterpillar versus non-caterpillar**						
Site	3	72.579	** < 0.001**	2014	1.099 ± 0.323	**0.001**
Year	2	1.831	0.400	2015	0.623 ± 0.319	0.053
Site × Year	6	16.536	**0.011**	2016	1.695 ± 0.290	** < 0.001**
**Other arthropods versus non-arthropods**						
Site	3	21.455	** < 0.001**	2014	0.928 ± 0.694	0.184
Year	2	7.033	**0.030**	2015	2.528 ± 0.635	** < 0.001**
Site × Year	6	9.447	0.150	2016	1.470 ± 0.565	**0.016**

(a) GLM models and the (b) linear contrasts between habitats (forest compared to urban habitat for each year; positive estimates indicate higher values in the forest). Statistically significant (*p* < 0.05) differences are highlighted in bold. For linear contrasts, *p* values were adjusted using the false discovery rate (FDR) method. Please note that the rows are for different information in parts (a) and (b).

Sample sizes (number of identified prey items, forest vs. urban):

Caterpillar versus non-caterpillar: 2014: 534 versus 279; 2015: 289 versus 398; 2016: 375 versus 376.

Other arthropods versus non-arthropods: 2014: 59 versus 86; 2015: 48 versus 120; 2016: 40 versus 164.

### Amount of nestling food

**Figure 3:**
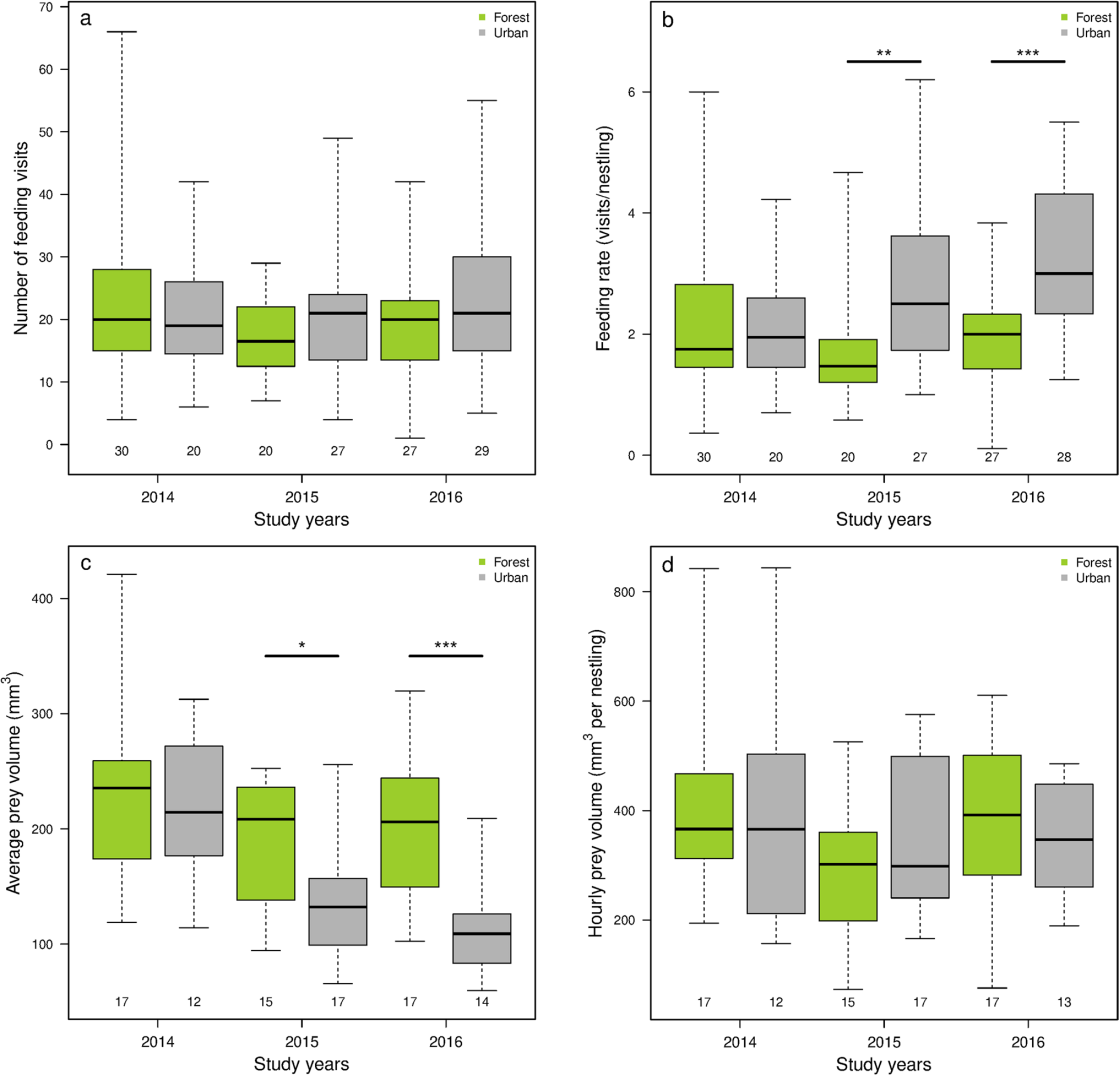
The amount of food provisioned to great tit nestlings during 60-min video observations in forest and urban habitats in the three years of the study. (a) Number of feeding visits of great tit parents (number/hour, both parents combined). (b) Feeding rate of parents per nestlings (number/nestling/hour). (c) Average prey volume delivered by the parents to nestlings (mm^3^/feeding visit). (d) Hourly prey volume per nestling, that estimates the total amount of food that one nestling received during the observation (mm^3^/nestling/hour). Boxes show the interquartile range, the thick line is the median, and whiskers refer to the range of data distribution. Sample sizes (number of broods) are indicated below each group. Statistically significant differences between the performance of forest and urban parents in a given year are marked with asterisks (*: *p* ≤ 0.05, **:*p* ≤ 0.01, *** *p* ≤ 0.001).

The total number of the parents’ feeding visits during the 60-min observations significantly differed between sites, but the site × year interaction was non-significant (Table 3a). Linear contrasts did not indicate a consistent habitat difference, meaning that on average urban parents visited their nests as frequently as forest parents in all years (Table 3b, Fig. 3a). However, when we compared feeding rates (i.e. the number of feeding visits per nestling) the linear model showed a significant effect for the site and also for the site × year interaction (Table 3a). The results of habitat contrasts showed higher feeding rates per nestlings for urban broods in 2015 and 2016 (Table 3b, Fig. 3b), but not in 2014.

**Table 3:** Comparison of feeding visits, feeding rates, and prey volumes between habitats.

(a) LM models				(b) Linear contrasts between urban and forest habitats in each year			
Predictors	DF	χ2	*p* value	year	contrast ± SE	t	adjusted *p* value
**Number of feeding visits**							
Site	3	1721.2	**0.001**	2014	− 0.592 ± 3.228	− 0.183	0.855
Year	2	139.1	0.517	2015	− 4.171 ± 3.292	− 1.267	0.311
Site × Year	6	863.4	0.231	2016	− 5.027 ± 2.806	− 1.791	0.226
**Feeding rate**							
Site	3	41.033	** < 0.001**	2014	− 0.312 ± 0.357	− 0.875	0.383
Year	2	5.388	0.126	2015	− 1.145 ± 0.364	− 3.149	**0.003**
Site × Year	6	16.950	**0.046**	2016	− 1.501 ± 0.315	− 4.772	** < 0.001**
**Average prey volume (mm**^**3**^**)**							
Site	3	74,677	** < 0.001**	2014	7.793 ± 24.623	0.317	0.752
Year	2	69,126	** < 0.001**	2015	59.331 ± 23.876	2.485	**0.023**
Site × Year	6	32,212	0.225	2016	92.924 ± 23.701	3.921	** < 0.001**
**Hourly prey volume (mm**^**3**^**)**							
Site	3	137,041	0.103	2014	− 40.627 ± 58.233	− 0.698	0.723
Year	2	106,500	0.090	2015	− 70.758 ± 56.467	− 1.253	0.642
Site × Year	6	331,497	**0.025**	2016	21.278 ± 59.906	0.355	0.723

Results of (a) LM models and the (b) derived linear contrasts (forest compared to urban habitat for each year; positive estimates indicate higher values in the forest) in the number of feeding visits (number/hour), feeding rate (number/nestling/hour), average prey volume (mm^3^/feeding visit), and hourly prey volume (mm^3^/nestling/hour). Statistically significant (*p* < 0.05) differences are highlighted in bold. For linear contrasts, *p* values were adjusted using the false discovery rate (FDR) method. Please note that the rows are for different information in parts (a) and (b).

Sample sizes (number of broods, forest vs. urban):

Number of feeding visits: 2014: 30 versus 20; 2015: 20 versus 27; 2016: 27 versus 29.

Feeding rate: 2014: 30 versus 20; 2015: 20 versus 27; 2016: 27 versus 28.

Average prey volume: 2014: 17 versus 12; 2015: 15 versus 17; 2016: 17 versus 14.

Hourly prey volume: 2014: 17 versus 12; 2015: 15 versus 17; 2016: 17 versus 13.

Regarding the average prey volume, we also found significant site and year effects, but the site × year interaction was not significant (Table 3a). Linear contrasts between habitats showed that in 2015 and 2016 urban parents brought smaller prey items per feeding visit than their forest conspecifics (Table 3b, Fig. 3c), whereas in 2014 the habitat difference was not significant. For hourly prey volume (i.e. the total amount of food that a nestling received during the 60-min observation), we found a significant site × year interaction (Table 3a). However, the habitat contrasts calculated from this model did not indicate significant habitat differences: on average urban and forest nestlings received the same amount of food during the observation period in all three years (Table 3b, Fig. 3d).

**Figure 4:**
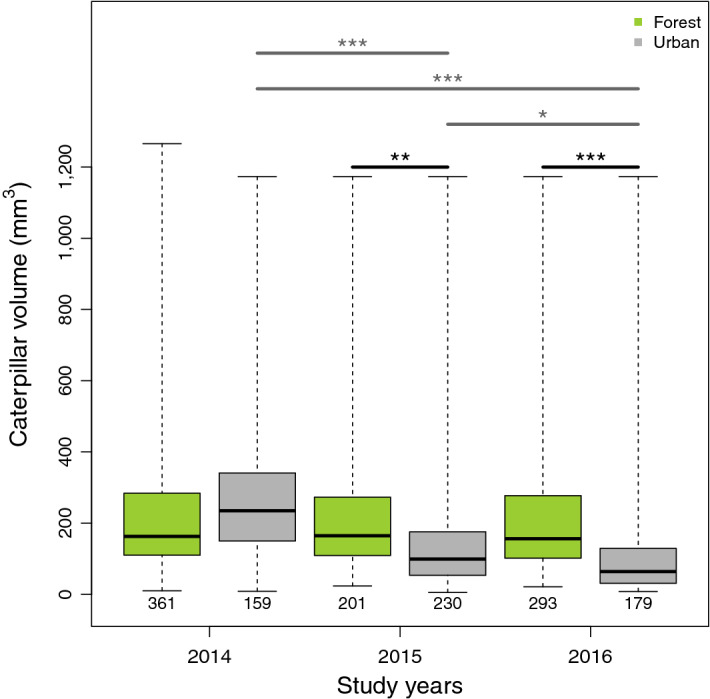
The volume of caterpillars provisioned to great tit nestlings in forest and urban habitats in the three years of the study. Sample sizes are indicated below each group. Boxes show the interquartile range, the thick line is the median, and whiskers refer to the range of data distribution. Statistically significant differences between habitats (black horizontal lines above the boxes) and years (grey horizontal lines above the boxes) are marked with asterisks (*: *p* ≤ 0.05, **:*p* ≤ 0.01, *** *p* ≤ 0.001).

For caterpillar volume, we found significant site and year effects and the site × year interaction was also significant (Table 4a). The linear contrasts testing the habitat difference revealed that in 2015 and 2016 urban parents delivered smaller caterpillars to their nestlings than forest parents (Table 4b, Fig. 4), whereas there was no difference in 2014 (Table 4b, Fig. 4.). Within the urban habitat, linear contrasts showed a significant between-year variation: caterpillars delivered to urban nestlings were significantly larger in 2014 than either in 2015 or 2016 (Table 4c, Fig. 4), and in 2015 they were also larger than in 2016 (Table 4c). In contrast, we found no annual difference in caterpillar size in the forest habitat (Table 4d, Fig. 4).

**Table 4:** Habitat and annual differences in caterpillar volume in the diet of great tit nestlings.

	DF	χ2	*p* value
**(a) LME model**			
Caterpillar volume			
Site	3	22.906	** < 0.001**
Year	2	29.180	** < 0.001**
Site × Year	6	21.847	**0.001**

year	contrast ± SE	t	adjusted *p *value
**(b) Habitat difference in caterpillar volume (linear contrast)**			
2014	− 0.201 ± 0.262	− 0.766	0.445
2015	0.730 ± 0.265	2.257	**0.010**
2016	1.203 ± 0.236	5.105	** < 0.001**
**(c) Annual difference in urban caterpillars’ volume (linear contrast)**			
2014–2015	1.150 ± 0.270	4.265	** < 0.001**
2014–2016	1.734 ± 0.263	6.589	** < 0.001**
2015–2016	0.584 ± 0.252	2.312	**0.022**
**(d) Annual difference in forest caterpillars’ volume (linear contrast)**			
2014–2015	0.220 ± 0.257	0.856	0.591
2014–2016	0.331 ± 0.234	1.415	0.478
2015–2016	0.111 ± 0.249	0.447	0.655

Results of (a) the LME models and (b-c-d) the derived linear contrasts. Section (b) shows the habitat contrasts in caterpillar volume for each year, positive estimates indicating higher values in the forest. Sections (c) and (d) show annual differences in urban and forest caterpillar volume, positive estimates indicating higher values in the first year of the comparison. Statistically significant (*p* < 0.05) differences are highlighted in bold. For linear contrasts, *p *values were adjusted by the false discovery rate (FDR) method.

Sample sizes (number of caterpillars, forest vs. urban):

2014: 361 versus 159; 2015: 201 versus 230; 2016: 293 versus 179.

## Discussion

Our results support, at least in some years, our initial predictions that (i) great tit nestlings in urban broods receive decreased proportions of caterpillars but increased proportions of non-arthropod food items (e.g. seeds), and that (ii) urban nestlings receive smaller prey items than nestlings in forest broods. However, our results did not support the prediction that (iii) urban nestlings receive a lower amount of food *per capita* because urban parents fed their nestlings more often relative to forest parents, which compensated for the smaller size of the prey items. Finally, as we expected (prediction iv), we found the smallest—and mostly non-significant—habitat difference in nestling diet in 2014 when the differences both in caterpillar biomass and in breeding success were also the smallest between urban and forest habitats^15^. Collectively, these results support the hypothesis that the shortage of optimal nestling food is an important limiting factor for the reproductive success of urban great tits^13–16^, similarly to those natural forest types that also have low caterpillar abundance^35,65^. Besides, our study provided novel evidence on the particular importance of food quality and highlighted remarkable annual variation in the level of high-quality food in urban environments.

### Nestling diet and reproductive success in ‘bad caterpillar’ years in urban habitat

In two of the studied years (2015, 2016), both the biomass of arboreal caterpillars and the breeding success of great tits were much lower in urban compared to forest habitats^15^. In these two years, caterpillars dominated the diet of forest nestlings (80–90%), whereas their proportion was much lower in urban nestlings’ food palette (50–70%). Urban parents compensated for the shortage of caterpillars by provisioning more non-arthropod food items (e.g. seeds, anthropogenic food) to their offspring. Despite having smaller broods, urban parents had to take a feeding effort (reflected by the total number of feeding visits per hour) similar to forest parents to provide a similar amount of food per offspring, due to the smaller size of prey items they could deliver. Thus, these results demonstrate that urban great tit parents are capable of quantitatively compensating for the low amount of food available in their environment by (1) taking care of broods of fewer offspring and (2) increasing their feeding effort per nestling.

Nevertheless, urban birds had much more reduced reproductive success in these years: they not only produced fewer fledglings (the mean habitat difference was 5.4 and 5.0 fledglings in 2015 and 2016, respectively), but the fledglings had also c. 20% lower body mass in urban compared to forest broods^15^ (on average by 3.4 and 3.3 g per nestling in 2015 and 2016, respectively; see Table 3 in the cited publication). Furthermore, Seress et. al^11^ also found that urban broods suffered from significantly higher starvation-related nestling mortality than forests broods. Thus, the above results on nestling body mass and survival indicate that even if urban parents can provide the same quantity of food per nestling, the unfavourable composition of nestling diet can clearly limit nestling development and survival in cities.

There are relatively few studies that examined both the composition and amount of nestling diet in relation to urbanisation. Two recent comprehensive studies conducted in the same populations of blue tits in Scotland partially support our conclusions. In line with our results, these studies found that urban nestlings received smaller proportions of caterpillars and the size of the caterpillars was smaller compared to the forest^31,32^. The feeding rate was almost twice as high in urban than in the forest broods in one study^31^, although it did not differ between the habitats in the other^32^. The overall conclusions of these studies were in line with ours: the low-quality nestling diet (lack of caterpillars) is responsible for the lower body mass of nestlings in urban broods^31,32^. Senar et al. (2021) also found that urban great tit nestlings received lower proportions of caterpillars, which was presumably the reason for the reduced breeding success in urban areas. However, they reported lower feeding rates in an urban habitat, although did not find habitat differences in the size of prey^66^. Mennechez and Clergeau (2006) found that in the common starling food volume received by the nestlings was reduced (cc. by 50%) in the urban centre compared to the periurban and suburban areas, and the composition of nestling food was also unfavourable in the urban centre (contained more human refuse)^39^. Several other studies that investigated only a single aspect of nestling food (i.e. either its composition or its quantity) similarly concluded that the low-quality nestling diet and/or the lower amount of nestling food is responsible for the reduced breeding success in urban habitats both in tit species^12,34,38,67,68^ and in other birds^3,5,40,43,69–71^.

### Nestling diet and reproductive success in a ‘good caterpillar’ year in urban habitat

In 2014 we found a much smaller habitat difference in nestling diet than in the other two years. Although urban nestlings received fewer caterpillars than forest nestlings, the non-caterpillar fraction of their food contained more other arthropods rather than non-arthropod food, similarly to the forest habitat. Furthermore, we did not find significant habitat differences in the amount of the nestling food: neither the hourly number of feeding visits and the feeding rate nor the average prey volume and hourly prey volume per nestling differed between the habitat types. In parallel with these results, the habitat differences in breeding success were also smaller than in the other two years^15^ (1.4 fledglings, and 1.6 g in their body mass; see Table 3 in the cited publication).

Our results on the annual changes in caterpillar size may provide a likely explanation for the reduced habitat difference in nestling diet and breeding success in 2014. This year urban pairs delivered much larger (2.2 times larger on average, calculated from raw data) caterpillars compared to the other two years, and similar in size to the caterpillars provisioned in the forest. Thus, urban parents were probably able to provide enough high-quality food for their nestlings, which resulted in improved nestling development. The importance of large prey items in the nestling diet was also reported in earlier studies. For example, Schwagmeyer & Mock estimated that a single large (> 2 cm) prey item’s dry weight is 30–40 times greater than that of a small one (< 0.6 cm). They also showed that the delivery rate of these large prey items (called ‘e-preys’) strongly predicted fledgling body mass and recruitment in house sparrows^36^.

It is not entirely clear why urban great tits could feed their young with larger caterpillars in 2014 than in the other two years. One possible explanation is that the unusually warm spring of 2014^15^ (see Fig. S2 in the cited publication) favoured the growth of caterpillars in the cities, as temperature can affect their feeding activity^72^. Our estimates for canopy caterpillar biomass do not support this explanation, however, because we did not detect increased caterpillar biomass in urban trees that year^15^. However, we did not monitor all tree species in our study sites, thus it is possible that large caterpillars represented moth species that developed on unmonitored but common tree species in our urban study sites. Similarly, we did not monitor caterpillars on other components of the vegetation including understory shrubs and grassy areas. In cities we often saw tits feeding on or searching for food on the ground or in the bushes, thus the increased caterpillar size in their diet may be due to the appearance and/or gradation of moth species that developed in these types of vegetation and reached large body size in 2014.

## Conclusions

In general, our results suggest that urban great tits are capable of delivering similar amounts of food per nestling as forest great tits due to the smaller brood size and because they feed their offspring relatively more often (compensating for the smaller amount of food per feeding event) than parents do in forests. Despite this, the size and survival of urban nestlings usually cannot reach the levels found in forest broods due to the lower quality nestling food (fewer and smaller caterpillars as well as increased proportions of non-arthropod food in the diet). However, our results from 2014 suggest that sometimes it is possible to catch a better year in the city when urban parents can feed their offspring with large caterpillars and relatively more other arthropods, which probably caused the detectable increase in urban birds’ reproductive success in that year. The crucial effect of the arthropod component of the nestling food was also supported experimentally in our study system: the provisioning of extra mealworms to urban nestlings during the chick-rearing period resulted in substantially larger body size and higher survival rates that were similar to their unsupplemented conspecifics in a forest habitat^16^.

Although urban areas may provide stable food availability across the year due to the large number of bird feeding stations, the nutrition value of these anthropogenic food sources is not suitable for rearing the nestlings (see Introduction). Therefore, one implication of our study is that increasing the abundance of phytophagous insects in cities would likely enhance the breeding success of urban insectivorous birds. A recent study on declining European butterfly populations suggested that simple measures such as reducing mowing intensity or creating new habitats by sowing wildflowers could produce quick wins^73^. Planting native trees at higher densities could also increase caterpillar populations and, indirectly, the reproductive success of insectivorous birds^74–76^. For example, Narango et al. found that native compared to non-native plants were more likely to host higher biomasses of caterpillars and that Carolina chickadees *(Poecile carolinensis)* strongly preferred to forage in native versus non-native plants during breeding^67,77^.

Finally, our study clearly demonstrates that long-term studies on urban populations with robust sample sizes are needed to fully understand the implications of among-year variation in environmental conditions. Our results suggest that caterpillar size, which is likely an important determinant of nestling diet quality, varies significantly among years in the cities, and this variation appears larger than in the forest (at least in the three years we studied). If data are based on only one year of observations and small sample sizes, the generalizability of the results can be very limited due to the strong annual variations^78^ like the one we reported for the nestling diet in this study.

## Supplementary Information

Supplementary Information.
