# Supplementary material for: Great tits feed their nestlings with more but smaller prey items and fewer caterpillars in cities than in forests
Source: Sci Rep. 2021 Dec 17;11:24161. doi: 10.1038/s41598-021-03504-4 (PMC8683465; doi:10.1038/s41598-021-03504-4)
Supplement: Supplementary file 1 — Supplementary Information. [file 41598_2021_3504_MOESM1_ESM.docx]

**Supplementary Material:**

**Great tits feed their nestlings with more but smaller prey items and fewer caterpillars in cities than in forests**

Csenge Sinkovics^1*^, Gábor Seress^1,2^, Ivett Pipoly^1,2^, Ernő Vincze^1,2,3^, András Liker^1,2*^

^1^  Behavioural Ecology Research Group, Center for Natural Sciences, University of Pannonia, PO Box 1158, 8210 Veszprém, Hungary

^2^ MTA-PE Evolutionary Ecology Research Group, University of Pannonia, PO Box 1158, 8210 Veszprém, Hungary

^3^ Theoretical Population Ecology and Evolution Group, Department of Biology, Lund University, Lund, Sweden

*Corresponding author: csenge.sinkovics@gmail.com, andras.liker@gmail.com

**Contents:**

- Supplementary Methods: page 2 – 4
- Figure S1 – S2: page 5 – 6
- Table S1 – S5: page 7 – 10

**Supplementary Methods: measuring prey size and prey volume from video recordings**

To estimate prey volumes, first we took a screenshot of each feeding event when a parent bird held the prey item in front of the nest box's entrance hole (Fig. S2). Then we used the software Fiji^1^ to measure the length and the average width of each prey item (in mm; excluding wings and legs of arthropods) using the vertical diameter of the nest box's entrance hole – which is always 32 mm in our nest boxes – as a size reference (Fig. S2). We measured the length of a food item along its fore-axis, while its width was estimated as the mean of three measurements conducted at each third along the prey’s length, as prey width can vary along the body of some prey types (Fig. S2). Because great tit parents are usually single prey loaders^2^, this method provides accurate and highly repeatable measurements on the size of nestling food items^3^ (intraclass correlation, repeatability within observer: ICC = 0.98, between observers: ICC = 0.93; see the cited publication for detailed validation of the method). We calculated prey volume (in mm^3^) assuming they had the shape of a cylinder^4^ using the following equation:

$$V={\pi l(0.5w)}^{2}$$

where *V* is the prey volume, and *l* and *w* are the length and average width of a prey item, respectively.

Our earlier study showed that with this method we can accurately measure both prey length and width for approximately one-third of the prey items^3^. However, in many of the remaining cases (i.e. when exact measurements on prey length and/or width were not possible from the screenshots) we were still able to estimate an approximate prey size relative to the birds’ beak length and height. For example, we were able to do this when a prey item was clearly visible in the bird’s beak, but we could not measure it because the parent did not stand right in front of the entrance hole (i.e. we could not use the hole’s diameter as an accurate size standard). To estimate prey size in these cases, we first created four length (short, medium, long, and extra-long) and three width categories (thin, medium, and thick) based on prey length and width relative to beak length and height, respectively, and categorized the visible but non-measurable prey dimension into one of these length and/or width categories (Table S4). Then we also classified all those preys into the same categories for which size dimensions (i.e. length and width) were measured accurately (i.e. in mm, see above; Table S4). From these latter, accurately measured length and width data, we calculated the mean for each of the four length and the three width categories (in mm; Average length and Average width columns in Table S4). Thus, using this procedure we were able to provide an estimate for the mean length or width for preys in each size categories, based on data of those subsets of preys in the categories that we were able to measure.

Finally, we also defined 12 volume categories as the combinations of the 4 length and 3 width categories (e.g. short × thin, short × medium, etc., Table S5). Using either the accurate size data (i.e. those measured in mm from screenshots) or the above-described size estimates for the length and width categories (Table S4), we calculated prey volume as follows:

1. When both length and width were accurately measurable for a prey item (in mm, from the screenshot) we calculated prey volume using the cylinder formula (see above).
2. If either the length or the width of the prey item was accurately measured but the other size parameter was only categorized, we assigned the category’s average value for that missing size parameter (Table S4), and calculated prey volume using this estimated size. For example, if a prey item was 20 mm long and its width was categorised as ‘thin’, we used the 20 mm length and the average value of the ‘thin’ category (2.48 mm) to calculate prey volume by the cylinder formula.
3. Finally, when neither the length nor the width could be measured, but we could categorize both parameters relative to the beak size, we used the estimated average volume of the volume category (Table S5). For example, for a prey in the short × thin category, this was 27.77 mm^3^ which is the average volume of this prey volume category.

In cases of (b) and (c), we used the average beak size (adult males and females combined) measured in our studied great tit populations (Sinkovics et al. 2018, unpublished data) as a size reference to define the upper and lower threshold of categories (there were no difference in beak sizes between the study populations, so we used the pooled average beak size). With the help of the above-described extrapolation method, we were able to estimate prey volume in 63.8 % of the prey items (62.5 % in urban, 65.3 % in forest broods), which proportion is similar to other studies investigating nestling diet^5^.

**References**

1. Schindelin, J. *et al.* Fiji : an open-source platform for biological-image analysis. *Nat. Methods* **9,** 676–682 (2012).

2. Kluijver, H. N. Daily routines of the Great Tit, Parus m. major L. *Ardea* **38,** 99–135 (1950).

3. Sinkovics, C., Seress, G., Fábián, V., Sándor, K. & Liker, A. Obtaining accurate measurements of the size and volume of insects fed to nestlings from video recordings. *J. F. Ornithol.* **89,** 165–172 (2018).

4. Slagsvold, T. & Wiebe, K. L. Hatching asynchrony and early nestling mortality: the feeding constraint hypothesis. *Anim. Behav.* **73,** 691–700 (2007).

5. Grieco, F. Time constraint on food choice in provisioning blue tits, Parus caeruleus: the relationship between feeding rate and prey size. *Anim. Behav.* **64,** 517–526 (2002).

6. QGIS Development Team. QGIS Geographic Information System. Open Source Geospatial Foundation Project. (2021).

7. Google. Lake Balaton, Hungary. Map data: Google, TerraMetrics. (2021).

8. Hydrographic map of Hungary. https://d-com/carte.php?num_car=2286&lang=en (2021). Available at: https://d-maps.com/carte.php?num_car=2286&lang=en.

**Figure S1.** The map of Hungary, indicating the urban (yellow) and forest (green) study sites and the number of nestboxes. The map was created with QGIS v. 3.20.1.^6^, the satellite image was retrieved from Google Maps (Google 2021)^7^ and the hydrographic map was downloaded from d-maps.com^8^.

**
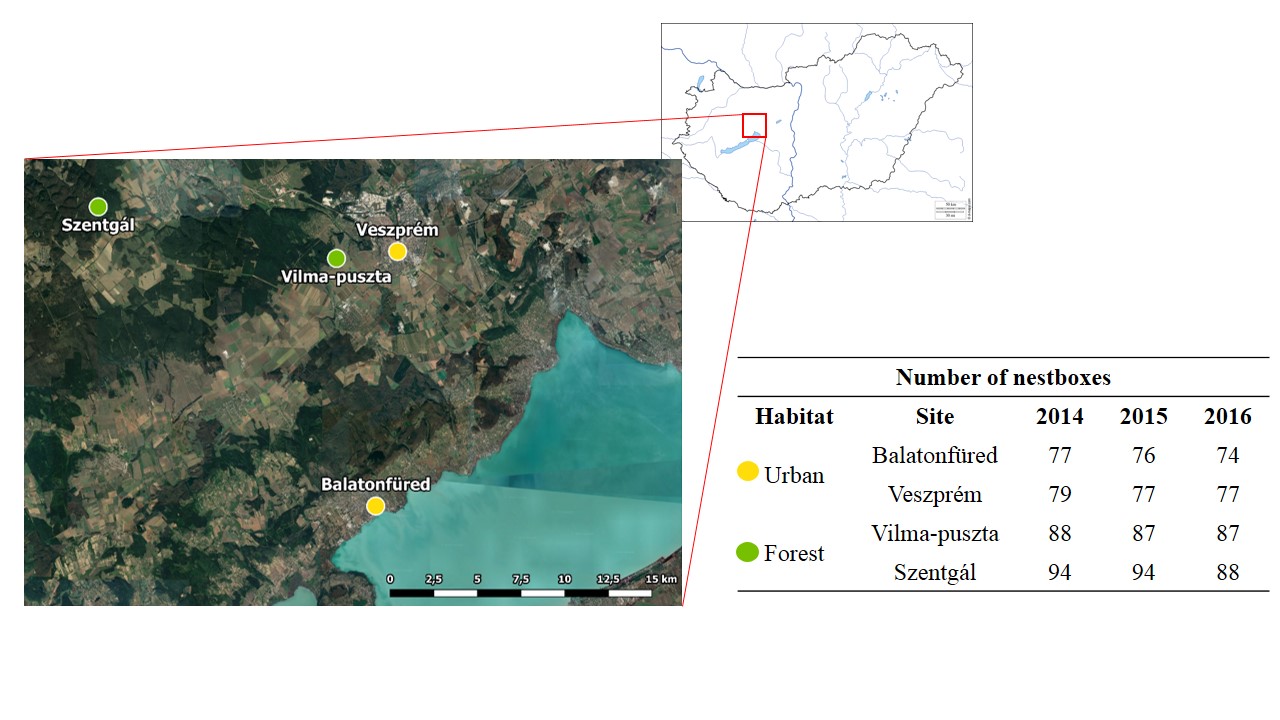
**

**Figure S2.** (a) Nest box with the shelf in front of the entrance hole and the black plastic box for the camera, as well as (b) the process of measuring prey size. The length of prey items was measured once along the middle axis (indicated by the white dotted line on the caterpillar), and prey width was measured three times along each third of the prey’s length (indicated by the black lines on the caterpillar). The white arrow shows the diameter of the entrance hole which was used as a size reference (note that it is the same on all of our nest boxes).


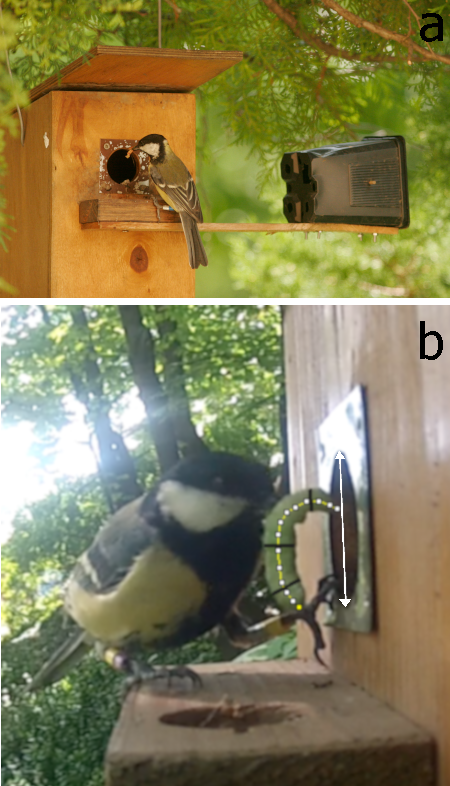


**Table S1**: Extended models related to the amount of nestling food. Statistically significant (p < 0.05) differences are highlighted in bold.

| **LM model** | | | |
| --- | --- | --- | --- |
|  | **DF** | **χ2** | **p value** |
| **Number of feeding visits** | | | |
| Site | 3 | 1389.2 | **0.006** |
| Year | 2 | 134.9 | 0.533 |
| Centered date | 1 | 97.6 | 0.34 |
| Time of the day | 2 | 234.9 | 0.335 |
| Temperature | 1 | 3.1 | 0.864 |
| Site × Year | 6 | 911.3 | 0.210 |
| **Feeding rate** | | | |
| Site | 3 | 37.609 | **< 0.001** |
| Year | 2 | 3.896 | 0.229 |
| Centered date | 1 | 0.598 | 0.500 |
| Time of the day | 2 | 0.153 | 0.943 |
| Temperature | 1 | 1.138 | 0.352 |
| Site × Year | 6 | 18.704 | **0.032** |
| **Average prey volume (mm^3^)** | | | |
| Site | 3 | 77323 | **< 0.001** |
| Year | 2 | 71969 | **< 0.001** |
| Centered date | 1 | 1 | 0.987 |
| Time of the day | 2 | 4173 | 0.581 |
| Temperature | 1 | 1168 | 0.582 |
| Site × Year | 6 | 32408 | 0.220 |
| **Hourly prey volume (mm^3^)** | | | |
| Site | 3 | 131934 | 0.103 |
| Year | 2 | 116914 | 0.065 |
| Centered date | 1 | 33102 | 0.209 |
| Time of the day | 2 | 77317 | 0.161 |
| Temperature | 1 | 155 | 0.931 |
| Site × Year | 6 | 345684 | **0.017** |

**Table S2.** Extended model related to the caterpillar volume. Statistically significant (p < 0.05) differences are highlighted in bold.

| **LME model** | | | |
| --- | --- | --- | --- |
|  | **DF** | **χ2** | **p value** |
| **Caterpillar volume** | | | |
| Site | 3 | 18.714 | **< 0.001** |
| Year | 2 | 27.237 | **< 0.001** |
| Centered date | 1 | 0.542 | 0.462 |
| Time of the day | 2 | 1.038 | 0.595 |
| Temperature | 1 | 0.257 | 0.612 |
| Site × Year | 6 | 21.554 | **0.001** |

**Table S3.** Extended models related to the composition of nestling diet. Statistically significant (p < 0.05) differences are highlighted in bold.

| **GLM model** | | | |
| --- | --- | --- | --- |
|  | **DF** | **χ2** | **p value** |
| **Caterpillar vs. non-caterpillar** | | | |
| Site | 3 | 61.285 | **< 0.001** |
| Year | 2 | 1.292 | 0.524 |
| Centered date | 1 | 0.91 | 0.34 |
| Time of the day | 2 | 2.805 | 0.246 |
| Temperature | 1 | 0.119 | 0.73 |
| Site × Year | 6 | 17.308 | **0.008** |
| **Other arthropods vs. non-arthropods** | | | |
| Site | 3 | 25.277 | **< 0.001** |
| Year | 2 | 9.131 | **0.010** |
| Centered date | 1 | 0.133 | 0.715 |
| Time of the day | 2 | 0.585 | 0.746 |
| Temperature | 1 | 2.510 | 0.113 |
| Site × Year | 6 | 10.823 | 0.094 |

**Table S4.** Size categories of prey items. We categorized the clearly visible but non-measurable prey items according to their sizes relative to the birds' beak, as given in the ‘Definition’ column. The corresponding size range is given in the ‘Size range’ column, as estimated from the average beak sizes of the studied great tit populations (Sinkovics et al., unpublished data). Accurately measured prey items (i.e. those for that we were able to measure length and width in mm) were also classified into the same categories, and the mean length and width of these preys are given in the ‘Average size’ column for each category.

| **Category** | **Definition** | **Thresholds by beak size** | **Average size** |
| --- | --- | --- | --- |
| **Length** | | |  |
| short | shorter than the beak (with at least one third of the beak length) | < 8.6 mm | 6.16 mm |
| medium | ~ beak length | 8.6-17.2 mm | 13.17 mm |
| long | longer than the beak (with at least one third of the beak length) | 17.2-25.8 mm | 21.19 mm |
| extra long | at least twice longer than the beak length | > 25.8 mm | 29.12 mm |
| **Width** | | |  |
| thin | thinner than the beak (with at least one third of the beak height) | < 3.2 mm | 2.48 mm |
| medium | ~ as thick as the beak | 3.2-6.3 mm | 4.13 mm |
| thick | thicker than the beak (with at least one third of the beak height) | > 6.3 mm | 7.03 mm |

**Table S5.** Prey volume categories based on the size categories relative to beak length, and their estimated average volume. The average volume was estimated from subsets of preys that fell into a given category and their size could also be measured accurately (in mm) from the screenshots.

| **Volume category** | **Average volume (mm^3^)** |
| --- | --- |
| short - thin | 27.77 |
| short - medium | 82.78 |
| short - thick | 256.81 |
| medium - thin | 64.14 |
| medium - medium | 175.67 |
| medium - thick | 472.03 |
| long - thin | 121.14 |
| long - medium | 284.16 |
| long - thick | 894.15 |
| extra long - thin | 182.8 |
| extra long - medium | 454.69 |
| extra long - thick | 1173.17 |
